# The Effects of Substance Abuse on Blood Glucose Parameters in Patients with Diabetes: A Systematic Review and Meta-Analysis

**DOI:** 10.3390/ijerph15122691

**Published:** 2018-11-29

**Authors:** Omorogieva Ojo, Xiao-Hua Wang, Osarhumwese Osaretin Ojo, Jude Ibe

**Affiliations:** 1 Department of Adult Nursing and Paramedic Science, University of Greenwich, London SE9 2UG, UK; 2 The School of Nursing, Soochow University, Suzhou 215006, China; wangxiaohua@suda.edu.cn; 3 University Hospital, Lewisham High Street, London SE13 6LH, UK; eseojo1@gmail.com; 4 Department of Family Care & Mental Health, University of Greenwich, London SE9 2UG, UK; J.C.Ibe@greenwich.ac.uk

**Keywords:** diabetes, substance abuse, opioids, fasting blood glucose, glycated haemoglobin, postprandial blood glucose, meta-analysis, systematic review, blood glucose parameters

## Abstract

*Background*: People who abuse substances are at increased risk of metabolic syndrome and diabetes resulting partly from increased cell damage and due to the effects of opioids on glucose homeostasis. Therefore, people with diabetes who abuse substances may carry greater health risks than the general population resulting from their effect on glucose metabolism. These substances may be in the form of cannabis, hallucinogens, opioids, and stimulants. Therefore, the aim of this review was to evaluate the effects of substance abuse on blood glucose parameters in patients with diabetes. *Method*: Databases including Embase, Psycho-Info, Google Scholar and PubMed were searched systematically for relevant articles from database inception to May 2018. Search terms including medical subject headings (MeSH) based on the Population, Intervention, Comparator and Outcomes (PICO) framework was used to access the databases. Eligible articles were selected based on set inclusion and exclusion criteria. The articles reviewed were evaluated for quality and meta-analysis and sensitivity analysis were carried out using the Review Manager (RevMan 5.3, The Cochrane Collaboration, Copenhagen, Denmark). The Random effects model was used for the data analysis. *Results*: Twelve studies which met the inclusion criteria were included in the systematic review, while nine articles were selected for the meta-analysis. The results of the meta-analysis showed that substance abuse does not have significant effects (*p* > 0.05) on postprandial blood glucose and glycated haemoglobin in patients with diabetes. With respect to the effect of substance abuse on fasting blood glucose, while this was significant (*p* < 0.05) following meta-analysis, the results of the sensitivity test did not demonstrate any significant difference (*p* > 0.05) between patients who abused substances compared with control. This would suggest that the effect of substance abuse on fasting blood glucose in these patients was not very reliable or not consistent. *Conclusions*: The effect of substance abuse on glycated haemoglobin and postprandial blood glucose in patients with diabetes was not significant. In the meta-analysis, while the value was slightly lower with respect to postprandial blood glucose, this was slightly higher in relation to HbA1c in the substance abuse group compared with control. On the other hand, the effect of substance abuse on fasting blood glucose was significant (*p* = 0.03) compared with control, but this was attenuated following a sensitivity test. A range of factors including eating habits, characteristics of drugs, erratic lifestyle of patients may explain the outcome of this review. There is the need for randomised controlled trials that will include diet and medication history in order to fully understand the effect of substance abuse on blood glucose parameters in patients with diabetes.

## 1. Introduction

The problem of substance abuse or illicit drug addiction is worldwide and it reduces productivity and places significant burden on healthcare systems while also negatively impacting the quality of life of individuals and communities [1]. The illicit drugs may include cannabis, hallucinogens, opioids, and stimulants [2]. Patients with diabetes frequently use opioids to manage diabetes-related neuropathic pain, which may put them at increased risk of opioid use disorder [2]. Chronic opiate use can result from opiate-related substance use disorder (SUD), or in the treatment of clinical opiate dependence [3]. People who abuse substances are at increased risk of metabolic syndrome and diabetes due partly to increased cell damage and lowered antioxidant potential of the cells [4]. It could also be due to the effects of opioids on glucose metabolism and opioid users’ poor diet choices [2]. Therefore, people with diabetes who abuse substances may carry greater health risks than the general population resulting from their effect on glucose metabolism [5]. In a study conducted in North Carolina, USA, adults with type 2 diabetes were found to have significantly higher rates of SUD (4.2%) compared with those without diabetes (2.1%) [2].

The effect of opium and other illicit substances on blood glucose parameters in patients with diabetes may be profound. In a review by Sharma and Singh Balhara [4], it was reported that among 49 patients with type 2 diabetes, it was found that the smoking of opium increased serum glucose and increased the risk of diabetic complications. In these patients who abuse opioid, the insulin secretory islet cells may not be responding appropriately to the glucose signals [4].

There have also been reported interactions between antidiabetic agents and prescribed, illicit and recreational drugs [5]. People who abuse substances are at greater risk of poorer diabetes outcomes including patient-related factors such as limited access to care, poor follow-up and suboptimal self-management skills [6,7,8]. There are also clinician factors including having negative views of patients with drug use and this may affect the quality of care that is provided for patients with diabetes [6].

There is evidence which shows that the use of illicit drugs including opioid is associated with glucose dysregulation, increased preference for sweet food, weight gain and the development of acute and chronic complications [2,9].

## 2. Why is the Review Important?

According to Sheldon and Quin [10], regular illicit drug use may hasten the onset of type 2 diabetes and it is associated with decreased insulin sensitivity. It has been shown that patients with co-occurring type 2 diabetes and illicit SUD do not comply as much with guidelines for laboratory testing (glycated haemoglobin) and clinical examinations (nephrology testing, eye examinations) and are more likely to experience lower-limb amputations and diabetes-related hospitalizations compared to patients with type 2 diabetes alone [2]. While substance use may play a role in type 2 diabetes outcomes, this is often overlooked from both research and clinical perspectives [2].

Therefore, the need for researchers to direct their interest in this area is becoming more important as the prevalence of diabetes is increasing globally [7]. A better understanding of approaches to managing SUD in patients with or at risk of type 2 diabetes may improve health outcomes in this high-risk population [2]. Areas of research interest could focus on determining the cause or directionality with respect to the high rate of type 2 diabetes in adults with opioid use disorder and the effect of substance abuse in patients with type 2 diabetes [2]. This is because there has been little research on the role of illicit SUD and diabetes outcomes [2].

The nature of illicit drug use and the often complicated lifestyles of those patients involved may contribute to the low level of research in this area and may make accurate assessment difficult [10]. However, the negative effects on morbidity, mortality and health economics suggest that it is a significant problem in diabetes practice [10]. The importance of managing SUD in patients with diabetes is underscored by the treatment guidelines recommended by the American Diabetes Association including limiting exposure to adverse lifestyle factors, the use of pharmacotherapies, managing psychosocial and behavioural factors, and treating comorbid conditions, including substance use disorders [2,7].

There are conflicting reports of the effect of substance abuse on blood glucose parameters including glycated haemoglobin (HbA1c) levels in patients with diabetes. While studies have indicated that nonopioid-dependent patients with diabetes demonstrate lower level of glycated haemoglobin compared with opioid-dependent individuals [11], there is evidence showing no significant change in the level of HbA1c in individuals with diabetes with or without opioid dependence [4,12]. However, in some Asian countries, opium is believed to have positive impact in lowering blood glucose [13].

Following a review of the literature, the authors of this review found no evidence of any systematic review and meta-analysis of the effects of substance abuse on blood glucose parameters in patients with diabetes. Therefore, the aim of this review is to evaluate the effects of substance abuse on blood glucose parameters in patients with diabetes.

Research Question: What effect does substance abuse have on markers of glycaemic control in patients with diabetes?

## 3. Method

This was a systematic review and meta-analysis conducted in accordance with established guidelines [14,15].

### 3.1. Data Sources and Search Strategy

Databases including Embase, Psycho-Info, Google Scholar and PubMed were searched systematically for relevant articles from database inception to May 2018. Search terms including medical subject headings (MeSH) based on the Population, Intervention, Comparator and Outcomes (PICO) framework (Table 1) was used to access the databases [14]. The search terms were combined using Boolean operators (AND/OR). All articles retrieved from the databases were exported to EndNote (Analytics, Philadelphia, PA, USA) for de-duplication.

### 3.2. Study Selection

**Figure 1:**
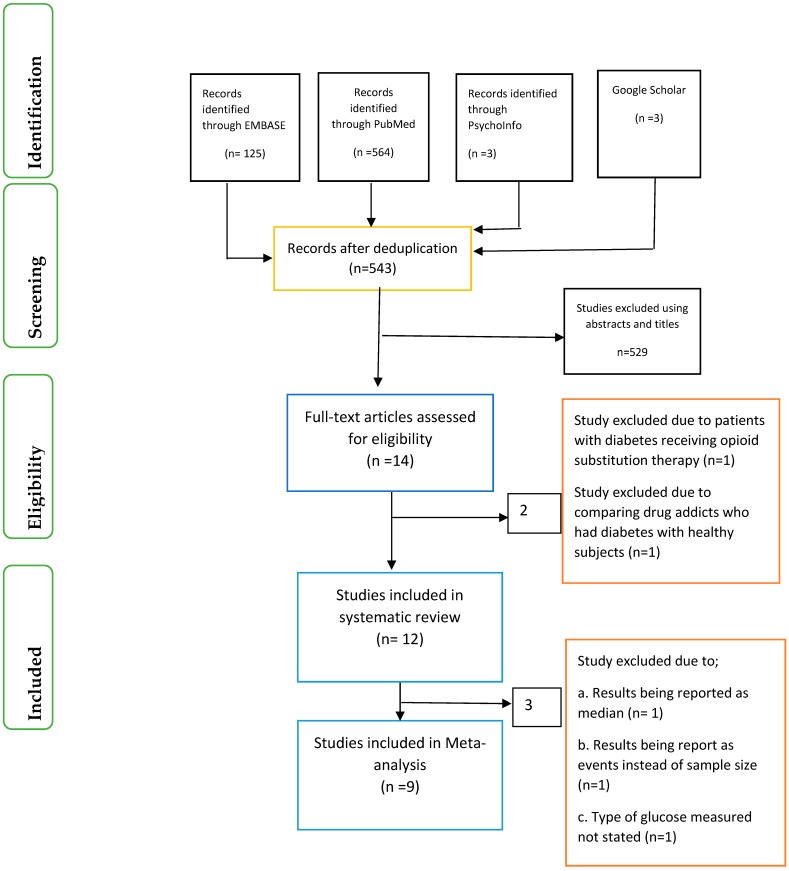
Prisma flow chart.

Identification of relevant articles, screening of articles using the abstracts and titles were conducted separately by the four researchers (O.O., X.-H.W., O.O.O. and J.I.) (Figure 1). In addition, eligibility of articles using the full-text was also based on a previously agreed inclusion and exclusion criteria by the four researchers. Differences were resolved among the researchers through consensus.

The records of articles accessed after de-duplication was 543. Following screening of the articles using the abstract and applying the exclusion and inclusion criteria, only 12 articles were eligible for the systematic review and nine articles for the meta-analysis.

### 3.3. Eligibility Criteria

The criteria for inclusion were: (1) Patients with type 1 or type 2 diabetes who are substance abusers; (2) Case-control study, Cross-sectional study and Retrospective cohort study; (3) Articles written in English. Exclusion criteria were: (1) Subjects with gestational diabetes; (2) Outcomes without blood glucose parameters.

### 3.4. Data Extraction

The data were extracted separately from the studies included by two researchers (O.O. and O.O.O.) and cross checked by two other researchers (X.-H.W. and J.I.). Differences among researchers were resolved by consensus. For this review, the following data were extracted (where available) from each study; country of study, length of study, study type/design, age of participants, gender and the type of substance abused. The blood glucose parameters of interest included were:

Fasting plasma glucose (mg/dL)
2-Hours Postprandial blood glucose (mg/dL)
Glycated haemoglobin (HbA1c) (%).

### 3.5. Quality Evaluation

The articles included in this review were evaluated using the Critical Appraisal Skills Programme (CASP) tool [16] and The Risk of Bias In Non-Randomised Studies of Interventions (RONINS-I) assessment tool [17]. The studies were evaluated in relation to bias due to confounding; selection of participants into the study; classification of interventions; deviations from intended interventions; missing data; measurement of outcomes; selection of the reported result [17]. The risk of bias could be classified as low, moderate, serious or critical [17]. One author (O.O.) assessed each study against the criteria in the assessment tool and this was cross-checked by other researchers (X.-H.W.; O.O.O. and J.I.). The assessments were based only on the data/information available in the studies.

### 3.6. Data Synthesis

The Mohammadali et al. [18] and Rahimi et al. [19] studies did not specify whether the data presented were expressed in the form of means ± standard deviation (SD) or means ± standard error of mean (SEM). The Rahimi et al. [19] study did not also specify the units of measurements. Therefore, we contacted the corresponding authors, but received no response. We then calculated the SD based on the means, sample sizes and the measures of dispersion and compared these with the results presented, and the findings were comparable. 

The Karam et al. study [11] presented the male and female results as separate findings and were thus included in the data analysis separately. Due to the limited number of studies involving patients with type 1 diabetes that were included in the meta-analysis, sub-group analysis could not be conducted for the type of diabetes. However, sub-group analysis was carried out for studies involving opium as SUD.

### 3.7. Statistical Analysis

The meta-analysis and sensitivity analysis were carried out using the Review Manager (RevMan 5.3, The Cochrane Collaboration, Copenhagen, Denmark) [20]. For sensitivity analysis, this was only conducted for parameters involving more than two studies. The random effects model was used for the data analysis of blood glucose because of the differences in the study designs of the various articles included and a forest plot provided a graphical representation for the different outcomes of interest. A *p* value of <0.05 was taken as statistically significant for the overall effect of the intervention. In addition, the statistic *I*^2^ on a scale of 0–100% was used as the measure of heterogeneity across the studies while a *p* value of 0.1 was used to determine statistical significance of heterogeneity.

## 4. Results

Twelve studies [11,12,18,19,21,22,23,24,25,26,27,28] met the inclusion criteria and were included in the systematic review (Table 2 and Table 3). Six of these studies [11,12,18,19,21,25] were conducted in Iran. Two studies were conducted in USA and one each was carried out in the UK, Yemen, Spain and Australia.

Most of the studies were either cross sectional studies (*n* = 5), retrospective cohort studies (*n* = 4) or case control studies (*n* = 3). While seven of the studies involved patients with type 2 diabetes, three studies had patients with type 1 diabetes and two studies did not state the type of diabetes of participants.

In addition, participants in six of the studies were abusing opium, whereas cocaine was the substance abused in three studies. One study each had Khat or different illicit drug abusers or the type of substance abused was not stated.

The findings of the different studies with respect to substance abuse were varied. According to Rezvanfar et al. [25], fasting blood glucose and HbA1c levels were lower in individuals with type 2 diabetes who abuse substances compared to non–abusers although the difference was not statistically significant. In contrast, Karam et al. [11] found that, HbA1c levels for males and females were higher in individuals with type 2 diabetes who abuse substances compared to non-abusers. Azod et al. [12] provided a different dimension when they revealed that while opium might decrease blood glucose temporarily, it had no clear and long-lasting effects on blood glucose and has no significant effect on HbA1c.

There were also no significant differences in serum levels of glucose between individuals with type 2 diabetes who abuse substances compared to non-abusers in the study by Mohammadali et al. [18] and in HbA1c between events shown and those not shown to be related to substance abuse [22]. Furthermore, Saif-Ali et al. [26] reported that chronic khat chewing does not affect serum glucose.

Therefore, it would seem that opium does not have significant effects on blood glucose regulation [19]. Instead, drug use appears to be a significant contributor to poor glycaemic control in young adults with type 1 diabetes [22] and cocaine users were found to have more frequent admissions for diabetic ketoacidosis [28].

In the case of patients with diabetes who were intravenous drug abusers, there were higher rates of diabetes complications [27] although Modzelewski et al. [24] observed that there was no association between active cocaine use at the time of hospital admission and development of hyperglycaemic crisis. Hosseini et al. [21] noted that, the use of opium for controlling blood glucose should not be encouraged. 

The studies included in this review did not provide data on the duration and quality of substance abused, diet history/compliance of participants and the type of diabetic medications except the study by Mohammadali et al. [18] that provided the antidiabetic medication history of patients and the Rezvanfar et al. [25] study that stated the mean duration (36 ± 6 months) of opium use.

### 4.1. Assessment of Risk of Bias of Included Studies

**Figure 2:**
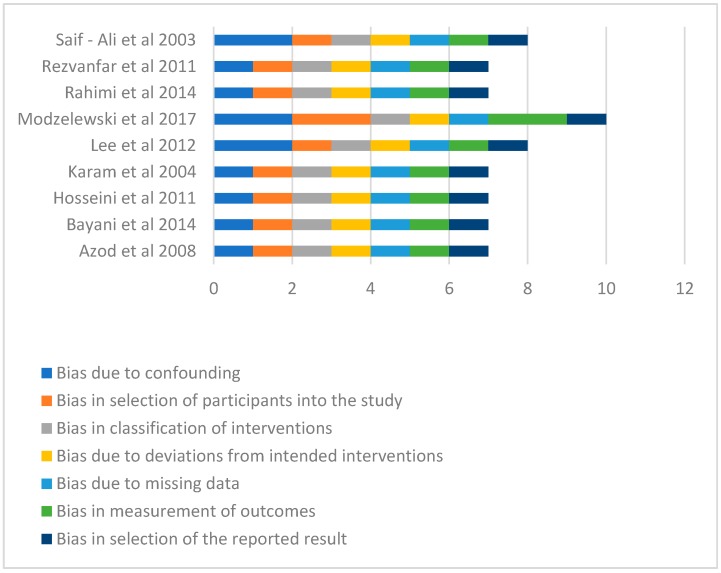
Summary of risk of bias (1 unit represents a low risk of bias while 2 represent moderate risk of bias).

**Figure 3:**
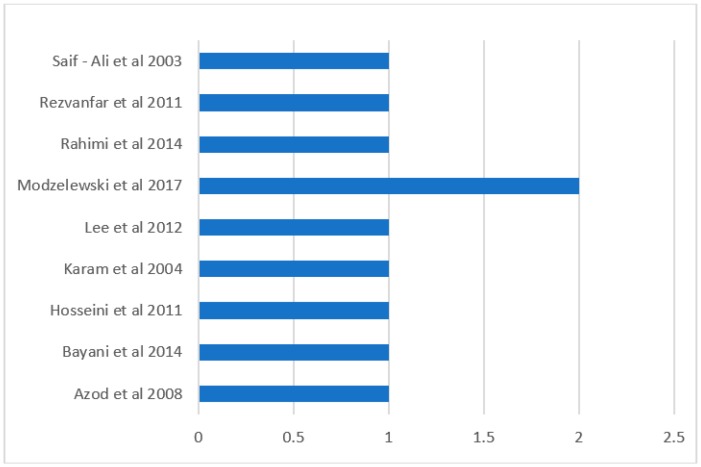
Overall Risk of Bias (1 unit represents a low risk of bias while 2 represent moderate risk of bias).

In terms of the risk of bias, the Modzelewski et al. [24] study demonstrated moderate risk of bias in respect of selection of participants into the studies and in the measurement of outcomes (Figure 2). All the other studies had low risk of bias in all the measures of assessment and had low overall risk of bias (Figure 2 and Figure 3). However, Modzelewski et al. [24] study’s overall risk of bias was moderate (Figure 3).

### 4.2. Effect of Substance Abuse on Fasting Blood Glucose

**Figure 4:**
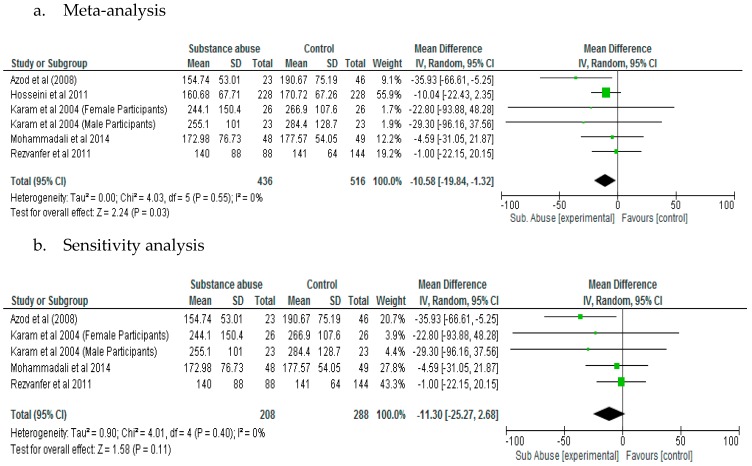
Results of Fasting Blood Glucose (mg/dL).

The results of the meta-analysis showed that substance abuse has significant effect (*p* = 0.03) on fasting blood glucose in patients with type 2 diabetes (Mean Difference, −10.58; 95% CI, −19.84, −1.32) (Figure 4a). However, a sensitivity analysis did not demonstrate significant difference (*p* = 0.11) between patients with diabetes who abused substances compared to those who did not with respect to fasting blood glucose (Figure 4b).

### 4.3. Effect of Substance Abuse on Postprandial Blood Glucose

**Figure 5:**
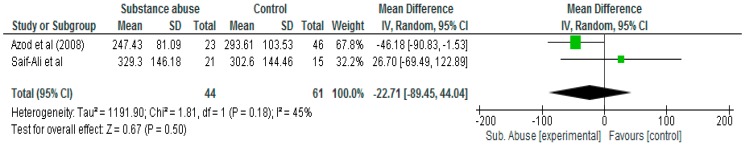
Results of Postprandial Blood Glucose (mg/dL).

There was no significant difference (*p* = 0.5) in postprandial blood glucose between patients with diabetes who abused substances compared with those who did not (Mean Difference, −22.71; 95% CI, −89.45, 44.04) (Figure 5).

### 4.4. Effect of Substance Abuse on Glycated Haemoglobin

**Figure 6:**
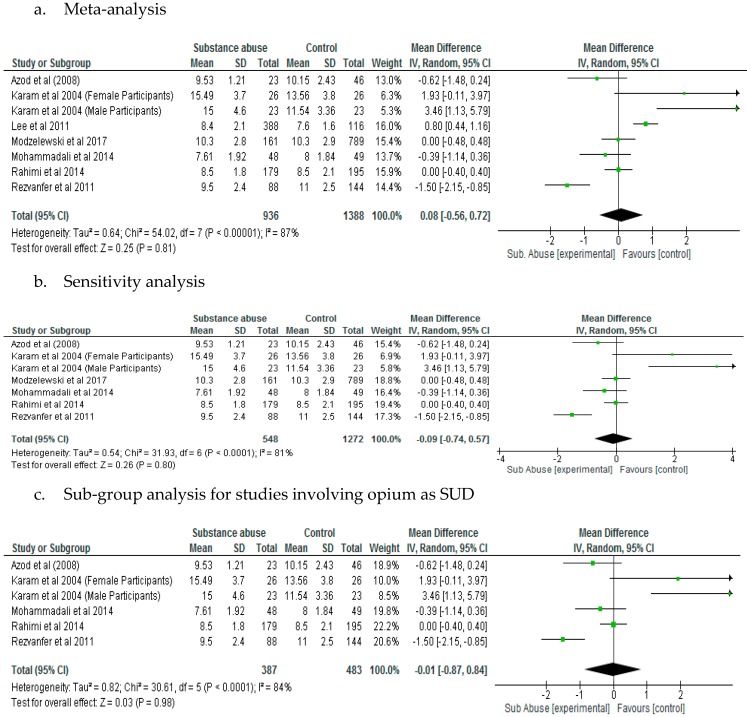
Results of Glycated Haemoglobin (%).

The effect of substance abuse on glycated haemoglobin was not significantly different between patients with diabetes who abused substances and control, following meta-analysis (*p* = 0.81). The Lee et al. [23] study which was the only study that had patients with type 1 diabetes included in the HbA1c analysis may have increased the level of heterogeneity (*I*^2^ = 87%). The Lee et al. [23] study also had highest weight. However, a sensitivity analysis which was carried out by removing the Lee et al. [23] study did not demonstrate significant difference (*p* = 0.80) in HbA1c levels between patients with diabetes who abused substances compared with control. Mean Difference was 0.08 for meta-analysis and −0.09 for sensitivity analysis (Figure 6a,b).

A sub-group analysis for studies involving opium as SUD showed that the mean difference between the substance abuse group and control with respect to glycated haemoglobin was −0.01 (−0.87, 0.84) and the difference was not significant (*p* = 0.98) (Figure 6c).

## 5. Discussion

The results of the meta-analysis have shown that substance abuse does not have significant effects (*p* > 0.05) on postprandial blood glucose and glycated haemoglobin in patients with diabetes. With respect to the effect of substance abuse on fasting blood glucose, while this was significant (*p* < 0.05) following meta-analysis, the results of the sensitivity test did not demonstrate any significant difference (*p* > 0.05) between patients who abused substances compared with control. This would suggest that the effect of substance abuse on fasting blood glucose in these patients was not very reliable or not consistent.

Although there appeared to be lower levels of fasting and postprandial blood glucose and higher level of HbA1c in the substance abuse group compared with control, these findings were not significant and/or were not consistent. The outcomes seem to confirm the results of the current systematic review which found that the effects of substance abuse on blood glucose parameters was variable in the different studies. In particular, while substance abuse was found to reduce fasting blood glucose and HbA1c in individuals with type 2 diabetes who abuse substances [25], it raised HbA1c in individuals with type 2 diabetes who abuse substances in another study [11]. Azod et al. [12] noted that although opium may reduce blood glucose, it does not seem to have a long-lasting effect on blood glucose. Both fasting and postprandial blood glucose reflect short term measures of blood glucose control while glycated haemoglobin represents the average glycaemia over a period of 2–3 months [29]. These findings appear to reinforce the challenges faced by researchers in trying to establish the cause and/or directionality between diabetes and substance use disorders and the controversy regarding the role of opioids in regulating glucose homeostasis [2,30]. In this regard, while there is evidence that opium consumption is the cause of impaired glucose tolerance and increased resistance to insulin, other studies have also shown severe hyperglycaemia following the discontinuation of opium [25].

### 5.1. Factors Contributing to the Observed Differences on the Effects of Substance Abuse on Blood Glucose Control

Sharma and Sing Balhara [4] observed that opioid dependent individuals are in a metabolic state that resembles diabetes although the causal hypothesis has not been fully established. The differences in the findings of the studies in this review may be partly due to differences in sample size and the sampling methods [19,25]. In addition, opium has about 20 alkaloids, therefore, their effects on metabolism and the endocrine system may be different compared to pure morphine, noscapine, and papaverine and the findings may thus, vary from one study to the other [11,30]. The differences in the findings may have also resulted from variations in the time of blood collection after the last consumption of drug, age range of subjects, duration of drug dependency, sociodemographic characteristics, the type of diabetes and its probable outcomes on function of liver and kidney [30]. Despite this position, only few associations were observed between the number of days of drug use and drug use severity in relation to blood glucose control in adult patients reporting recent drug use [6]. In the same study, cocaine use appeared to be associated with worse blood glucose control than marijuana. The action of cocaine on alpha—adrenergic system which may lead to hyperglycaemia has been reported as a possible mechanism of action of cocaine on blood glucose homeostasis [9].

Patients with diabetes who abuse substances may have poorer glycaemic control due to a number of reasons including their poor nutrition state, mental illness and erratic lifestyle such as poor compliance with prescribed medications including insulin and poor diabetic clinic attendance [10,31,32]. In fact, the effect of poor medication management in patients with diabetes who abuse substances could be bi-directional. For example, an overdose of insulin could lead to hypoglycaemia while insufficient dose of the same medication could cause hyperglycaemic episode irrespective of the underlying effect of substance abuse [33]. Opioid use can lead to sedation and cognitive decline which could affect the patients’ ability to provide selfcare, access healthcare provisions including diabetic tests and the potential for increased diabetes related complications [2].

Therefore, in order to assess the effect of drug use on blood glucose parameters in patients with diabetes who abuse substances, it is essential to have information on the characteristics of the drugs being abused and studies should not just be limited to whether the patients are abusing drugs or not [6]. This is because the frequency, the type of drug and/or its severity may have different effects on clinical outcomes including blood glucose parameters of patients, although these data are difficult to obtain [6] and many of the studies in this review did not report this kind of information. In this regard, substance abuse may contribute to the non compliance with diabetic medication including insulin [9].

### 5.2. The Increase in the Risk of Hyperglycaemia in Patients with Diabetes Who Abuse Opioids

According to Sharma and Sing Balhara [4], there appears to be evidence which point to the role of opioids in glucose homeostasis. While there was evidence of some effects of SUD on glycaemia in patients with diabetes based on the current meta-analysis, these were not significant with respect to glycated haemoglobin and postprandial blood glucose although the effect on fasting blood glucose was significant albeit not consistent. In a previous review [2], high rates of type 2 diabetes (25–28%) in adults who either previously or were receiving opioid maintenance therapy was reported. The findings of higher prevalence of patients with type 2 diabetes in individuals who abuse opioids may be due to the effect of opioids on glucose metabolism and the poor diet choices of opioid users [2]. Data from studies have found that the use of opioids is associated with glucose dysregulation and worse diabetes performance measures [2,34]. The mechanisms of action of opioids including morphine on blood glucose homeostasis have been demonstrated in human studies and in animal models. These include increased production of adrenocorticotrophic hormone (ACTH) and plasma adrenaline, nor-adrenalin, cortisol and glucagon [9,11,19]. These are counter-regulatory hormones and their actions oppose that of insulin in the facilitation of glucose into the cells [19]. According to Azod et al. [12], in animal studies, the hyperglycaemic effect of morphine may be due to its effect on glucagon production without corresponding release of insulin. The acute hyperglycaemic effect of morphine may be controlled by the central nervous system causing decreased insulin secretion and glycogenolysis [13]. In patients with diabetes, this is exacerbated due to increased glucagon production [13].

It has been observed that patients who are dependent on opioids do not respond adequately to insulin signals thus leading to loss of glucose homeostasis [4]. However, chronic heroin administration can produce a state of fasting hyperinsulinaemia even in the absence of glucose intolerance and may interfere negatively with carbohydrate metabolism [35]. The hypothesis that has been suggested is based on the possible development of fasting hyperglycaemia as a result of either insulin resistance through a secondary down-regulation of insulin receptors or accelerated beta cell failure [35]. However, the challenge of confirming this hypothesis reside on a number of factors including narcotic withdrawal, intercurrent infections, eating habits and early death [35].

### 5.3. The Lowering of Glycaemic Parameters in Patients with Diabetes Who Abuse Opioids

It has also been reported that morphine could exert a hypoglycaemic effect at low dose infusion when the endocrine pancreatic function is fixed at basal level [12]. The authors noted that the findings relating to the hypoglycaemia was independent of detectable changes in insulin, glucagon, epinephrine and suggested that the hypoglycaemic effect of morphine results from the interaction of the opiate with non-mu receptors, either in the liver or the central nervous system.

Another possible explanation in the lowering of postprandial blood glucose in patients with diabetes who abuse substances may be due to decrease in gastric emptying resulting from opioid µ-receptor activation and delay in intestinal glucose absorption [13]. According to Kouros et al. [30], increased utilisation of glucose and a decrease of hepatic gluconeogenesis resulting from the activation of peripheral opioid µ-receptors and alterations of glucose metabolism which may be related to genes have been proposed as possible mechanisms involved in decreasing the plasma glucose. The effects of medication taken by patients to manage their condition and other co-morbidities may also contribute to the lowering of blood glucose in patients with diabetes who abuse substances.

### 5.4. The Pharmacological Effects of SUD on Glucose Management Versus Non-Pharmacological Effects of SUD

Patients with diabetes often use opioids to manage diabetes related pains including peripheral neuropathic pain [1,36]. About 16–20% of patients with diabetes experience chronic neuropathic pain [36]. However, the long term treatment with opioids may lead to opioid abuse which could have impact on glucose homeostasis [2].

In terms of illicit substance use disorder, it would appear that patients with co-occurring type 2 diabetes and illicit SUD are more likely to have lower limb amputation and diabetes related complications compared with those with type 2 diabetes alone [2].

## 6. Limitations

Most of the studies included in this review were carried out in Iran, therefore, this may affect its broader application. In addition, most of the studies did not provide data on the duration and quality of substance abused, diet history/compliance of participants and the type of diabetic medications thus making analysis difficult. The Mohammadali et al. [18] and Rahimi et al. [19] studies did not specify whether the data presented were expressed as means ± SD or means ± SEM. Although the SD was calculated based on the means, sample sizes and measures of dispersion and were found comparable to the results presented, the failure to properly report these measures is a limitation of those studies.

## 7. Conclusions

The effect of substance abuse on glycated haemoglobin and postprandial blood glucose in patients with diabetes was not significant. However, while the value was slightly lower with respect to postprandial blood glucose, this was slightly higher in relation to HbA1c in the substance abuse group compared with control. On the other hand, the effect of substance abuse on fasting blood glucose was significant (*p* = 0.03) compared with control, but this was attenuated following a sensitivity test. This would suggest that the effect of substance abuse on fasting blood glucose is not very reliable or is transient. A range of factors including narcotic withdrawal, intercurrent infections, eating habits, characteristics of drugs, erratic lifestyle of patients may explain the outcome of this review. There is the need for randomised controlled trials that will include diet and medication history in order to fully elucidate the effect of substance abuse on blood glucose parameters in patients with diabetes.

## Figures and Tables

**Table 1:** Search Terms and Search Strategy.

Patient/Population	Intervention	Comparator	Outcomes of Interest	Combining Search Terms
Patients with diabetes	Substance Abuse		Outcomes of interest	
Type 2 diabetes OR Type 1 diabetes OR Diabetes complications OR Diabetes mellitus, type 2 OR Diabetes mellitus, type 1 OR Diabetes mellitus	Substance-Related Disorders OR substance * OR Marijuana Abuse OR Amphetamine-Related Disorders OR Cocaine-Related Disorders OR Opioid-Related Disorders OR opiate * OR opioid * OR Heroin Dependence		Glycated hemoglobin OR Fasting blood glucose OR Post-prandial blood glucose OR Fasting insulin OR Fructosamine	Column 1 AND Column 2 AND Column 3

* (Truncation symbol).

**Table 2:** Characteristics of the articles included in this review (*N* = 12).

Study Reference	Country	Length of Study	Study Type/Design	Sample Size/Description	Age	Gender	Diabetes Type	Type of Substance Abused
Azod et al., 2008 [12]	Iran	No data	Cross-sectional study	23 opium 46 non-opium	Mean 55.24–60.52 years	No data	Type 2 DM	Opium
Hosseini et al., 2011 [21]	Iran	2008–2010	Cross-sectional study	228 opium 228 non-opium	Mean 58.9 (SD = 9.2 years)	92% male	91% were type 2 DM	Opium
Isidro & Jorge 2013 [22]	Spain	2005–2009	Retrospective cohort study	52 events with substance use; 201 events without substance use	Mean 29.5–36.7 years	58% male	Mostly Type 1 DM (83.8–92.3%)	Cocaine & polydrug use
Karam et al., 2004 [11]	Iran	No data	Case-control study	23 male and 26 female opium 23 male and 26 female non-opium	35–65 years	53% female	Type 2 DM	Opium
Lee et al., 2012 [23]	Australia	No data	Cross-sectional survey	388 substance users 116 non-users	Mean 30–32 years	63–79% female	Type 1 DM	Different illicit drugs
Modzelewski et al., 2017 [24]	USA	2004–2010	Retrospective case-control analysis	161 DM patients with cocaine use; 789 DM without drug use	Mean 47.3 years	66% male	No data	Cocaine
Mohammadali et al., 2014 [18]	Iran	2006–2007	Cross-sectional study	48 opium users 49 non-opium users	Mean 64 years	>60% female	Type 2 DM	Opium
Rahimi et al., 2014 [19]	Iran	No data	Cross-sectional study	179 opium users 195 non-opium users	Mean 53.5–58.2 years	No data	Type 2 DM	Opium
Rezvanfar et al., 2011 [25]	Iran	2009–2010	Case-control study	88 opium users 144 non-opium users	Mean 55–57 years	All male	Type 2 DM	Opium
Saif-Ali et al., 2003 [26]	Yemen	No data	Case-control study	21 khat chewers 15 non Khat chewers	25–65 years	All male	Type 2 DM	Khat
Saunders et al., 2004 [27]	UK	1997–2002	Retrospective case note analysis	9 intravenous drug users 18 non intravenous drug users	Mean 33 years	>75% male	Type 1 DM	No data
Warner et al., 1998 [28]	USA	1985–1994	Retrospective Case-control study	27 cocaine user 85 non cocaine user	Mean 28.2–29.7 years	>65% female	No data	Cocaine

Abbreviations: DM (Diabetes Mellitus); SD (Standard deviation).

**Table 3:** Blood glucose parameters among individuals with diabetes based on their substance use status.

Study Reference	Participants Studied	Fasting Blood Sugar	2-Hrs Postprandial Blood Glucose	Random Blood Sugar	Glycated Haemoglobin
Azod et al., 2008 [12]	Substance abusers	154.74 mg/dL (SD = 53.01)	247.43 mg/dL (SD = 81.09)	No data	9.53% (SD = 1.21)
	Non-substance abusers	190.67 mg/dL (SD = 75.19) *p* = 0.04	293.61 mg/dL (103.53) *p* = 0.06	No data	10.15% (SD = 2.43) *p* = 0.25
Hosseini et al., 2011 [21]	Substance abusers	160.68 mg/dL (SD = 67.71)	No data	No data	No data
	Non-substance abusers	170.72 mg/dL (SD = 67.26) *p* = 0.117	No data	No data	No data
Isidro & Jorge 2013 [22]	Substance abusers	No data	No data	32.8 mmol/L (SD = 14.5)	11.4% (SD = 1.8)
	Non-substance abusers	No data	No data	30.5 mmol/L (SD = 13.6) *p* (No data)	11.6% (SD = 2.2) *p* (No data)
Karam et al., 2004 [11] (for men)	Substance abusers	14.17 mmol/L (SEM = 1.17)	No data	No data	15.00% (SEM = 0.96)
	Non-substance abusers	15.8 mmol/L (SEM = 1.49) *p* = 0.4650	No data	No data	11.54% (SEM = 0.7) *p* = 0.0094
Karam et al., 2004 [11] (for women)	Substance abusers	13.56 mmol/L (SEM = 1.64)	No data	No data	15.49% (SEM = 0.72)
	Non-substance abusers	14.83 mmol/L (SEM = 1.17) *p* = 0.5700	No data	No data	13.56% (SEM = 0.75%) *p* = 0.0915
Lee et al., 2012 [23]	Substance abusers	No data	No data	No data	8.4% (SD = 2.1)
	Non-substance abusers	No data	No data	No data	7.6% (SD = 1.6) *p* = 0.03
Modzelewski et al., 2017 [24]	Substance abusers	No data	No data	480.9 mg/dL (SD = 211.8)	10.3% (SD = 2.8)
	Non-substance abusers	No data	No data	442.1 mg/dL (SD = 226.2) *p* = 0.045	10.3% (SD = 2.9) *p* = 0.903
Mohammadali et al., 2014 [18]	Substance abusers	172.98 mg/dL (SD = 76.73)	No data	No data	7.61% (SD = 1.92)
	Non-substance abusers	177.57 mg/dL (SD = 54.05) *p* = 0.787	No data	No data	8.0% (SD = 1.84) *p* = 0.168
Rahimi et al., 2014 [19]	Substance abusers	172.1 mg/dL (SD = 73.1)	No data	No data	8.5% (SD = 1.8)
	Non-substance abusers	177.6 mg/dL (SD = 66.1) *p* = 0.440	No data	No data	8.5 (SD = 2.1) *p* = 0.970
Rezvanfar et al., 2011 [25]	Substance abusers	140.0 mg/dL (SD = 88.0)	No data	No data	9.5% (SD = 2.4)
	Non-substance abusers	141.0 mg/dL (SD = 64.0) *p* = 0.71	No data	No data	11.0% (SD = 2.5) *p* = 0.006
Saif-Ali et al., 2003 [26]	Substance abusers	No data	329.3 mg/dL (SEM = 31.9)	No data	No data
	Non-substance abusers	No data	302.6 mg/dL (SEM = 37.3) *p* (No data)	No data	No data
Saunders et al., 2004 [27]	Substance abusers	No data	No data	No data	Median 10.2% (IQR = 1.96)
	Non-substance abusers	No data	No data	No data	Median 9.1% (IQR = 2.34) *p* = 0.061
Warner et al., 1998 [28]	Substance abusers	No data	No data	593.4 mg/dL (SD = 238.9)	No data
	Non-substance abusers	No data	No data	531.1 mg/dL (SD = 185.8) *p* < 0.05	No data

Abbreviations: IQR (Inter Quartile Range); SD (Standard deviation); SEM (Standard Error of Mean).
